# The strength of soil-plant interactions under forest is related to a Critical Soil Depth

**DOI:** 10.1038/s41598-019-45156-5

**Published:** 2019-06-14

**Authors:** Philipp Goebes, Karsten Schmidt, Steffen Seitz, Sabine Both, Helge Bruelheide, Alexandra Erfmeier, Thomas Scholten, Peter Kühn

**Affiliations:** 1 Institute of Geography, Soil Science and Geomorphology, University of Tübingen, Rümelinstrasse 19-23, Tübingen, Germany; 2 Institute of Biology, Geobotany and Botanical Garden, Martin-Luther University Halle-Wittenberg, Am Kirchtor 1, Halle, Germany; 3 Environmental and Rural Science, University of New England, Armidale, NSW 2351 Australia; 4 Institute for Ecosystem Research, Kiel University, Olshausenstrasse 75, Kiel, Germany; 5 German Centre for Integrative Biodiversity Research (iDiv) Halle-Jena-Leipzig, Deutscher Platz 5e, 04103 Leipzig, Germany

**Keywords:** Forest ecology, Biodiversity, Environmental impact

## Abstract

Soil properties and terrain attributes are of great interest to explain and model plant productivity and community assembly (hereafter P&CA). Many studies only sample surface soils, and may therefore miss important variation of deeper soil levels. We aimed to identify a critical soil depth in which the relationships between soil properties and P&CA were strongest due to an ideal interplay among soil properties and terrain attributes. On 27 plots in a subtropical Chinese forest varying in tree and herb layer species richness and tree productivity, 29 soil properties in six depth columns and four terrain attributes were analyzed. Soil properties varied with soil depth as did their interrelationships. Non-linearity of soil properties led to critical soil depths in which different P&CA characteristics were explained best (using coefficients of determination). The strongest relationship of soil properties and terrain attributes to most of P&CA characteristics (adj. R^2^ ~ 0.7) was encountered using a soil column of 0–16 cm. Thus, depending on the biological signal one is interested in, soil depth sampling has to be adapted. Considering P&CA in subtropical broad-leaved secondary forests, we recommend sampling one bulk sample of a column from 0 cm down to a critical soil depth of 16 cm.

## Introduction

Soils provide nutrients, water and space for trees, herbs and other plants and thus are the basis for life on earth^1^. Moreover, they play an important role for ecosystem functioning^2^. Soils differ in their properties, which can be distinguished at a time scale relevant for ecological processes in stable components such as texture or mineral composition and dynamic characteristics such as nutrient contents or soil pH of humus content^3^. While the former are largely independent from vegetation, the latter are affected by plants, and in turn, exert influence on plant growth and species composition (e.g.^4,5^). The distinction between stable and dynamic soil properties goes back to the concept of soil quality assessment^3,6^. On the one hand, dynamic soil properties (e.g. soil pH, nutrient contents, base saturation) can be considered properties that may change relative quickly (within years or decades) related to biological processes, vegetation cover and management practices^7^. However, species-rich tropical and subtropical forests tend to show these feedbacks to soil only to a minor degree as litter inputs are homogenized by high diversity^8^. On the other hand, stable soil properties (e.g. grain size distribution, mineralogical composition) may change over millennia or longer periods of time and can be considered as part of the basic site properties which barely change due to biotic activities at ecological time scales^9^.

As soil formation is predominantly a vertical process related to climate, parent material, relief, organisms, time and spatial position^10^ and as weathering and humus input is strongest at the surface and decreases with depth^11^, most soil properties are depth-dependent^11–13^. Processes such as acidification, calcification or loamification favor these depth-dependent gradients in soil chemical and physical properties. For instance, the process of loamification leads to smaller soil particle sizes while acidification favors acid transfer in deeper depths. Exceptions may be soils affected by turbation processes (e.g. cryoturbation in permafrost affected soils)^14,15^, shrink and swell behavior^16,17^ or transport processes along slopes (e.g. soils affected by water and wind erosion and sedimentation)^18,19^. In general, depth gradients in soil chemical and physical properties are influenced by biogenic, geogenic and pedogenic processes^20,21^. The deeper the soil layers are located, the lower is the influence of plant cover and soil biota and the higher is the influence of weathering, leading to specific depth functions for soil properties^12,22,23^. For instance, soil organic carbon (SOC) decreases with increasing soil depth as organic material enters the soil predominantly from the top and then subsequently is incorporated into deeper soil layers^24,25^. This is the case as well for elements such as Ca, Mg, Na or K, originating from the parent material in an early successional stage and entering plant nutrient cycles in mature stages. Those nutrients, now bound to organic matter, can then be decomposed e.g. within the leaf litter layer or deadwood and burrowed into the soil being diluted with increasing depths. In contrast, in the course of weathering, the proportion of coarse material (CM) of the bedrock decreases with increasing distance to the parent material, if the soils are not layered^26^. As the relationship of all soil characteristics with depth is mainly non-linear^27,28^, it should be expected that interrelations between different soil properties change with soil depth^23^, and may have effects on biotic components. In addition, this non-linearity with soil depth complicates the processes involved^29^.

Because of the mutual influence of soil properties and biotic components, there are interaction pathways in both directions. Soil properties act as filters for organisms (community assembly) and as drivers of biotic functions (productivity) in terrestrial ecosystems. Plants preferably use an ecological optimum of those soil properties in which functioning and growth is enhanced^5,30,31^. It is clear that abiotic site conditions affect individual tree growth^32,33^. In addition, higher C, N and P in tropical forest soil were found to be strongly correlated with higher tree species richness, while those nutrients were negatively correlated with tree density^34^. Thus, nutrient availability can affect tree species richness and composition on larger scales^8^. Moreover, pH influences uptake of specific nutrients (Ca, Mg, K in basic, Al and Mn in acidic environments) by plants^8^. Another study from Southeast Asia reported that tree diversity was negatively correlated with elevation, coarse material and C whereas it correlated positively with CN ratio and pH^35^. However, no distinction between different soil depth increments was made in the mentioned studies.

We hypothesize that there is a depth section from the topsoil down to a specific soil depth that maximizes the predictability of tree and herb layer growth and functioning from soil properties. Soil data extracted from this depth increment might be superior in explaining the relationships with productivity and community assembly (hereafter P&CA) compared to all other soil data of deeper or shallower depth increments. Thus, we introduce the novel term “critical soil depth” that defines its lower boundary as the maximum depth of sampling to gain optimal results. This concept of a critical soil depth has rarely been in focus of research, and if, mostly as a byproduct dealing with depth-specific feedbacks of plants. In Scotland, Baddeley and Watson showed that 10 cm is the most suitable depth for predicting root survivorship of *Prunus avium*. Yet, depth-specific feedbacks of six tree species revealed positive and negative effects for C and N in temperate forests^36^. In addition, plant community composition was affected by substrate depth^37^ and survivorship of plants increased with substrate depth^38^. However, authors criticize the lack of attention that is given to soil properties in different depths in most root-soil feedback studies^39^. In general, the plant-soil feedback is an important mechanism that can both maintain and explain species diversity and abundance^40^. In addition, a distinction between herb and tree layer based on different rooting depths may be needed to model and understand the relationship of soil properties to P&CA properly.

Terrain variation (slope, aspect, elevation) also causes different soil properties, even if the soil is derived from the same parent material^8,41,42^. In addition, plant growth strongly depends on water and light resources which can be derived from slope, aspect and elevation^43,44^. Legendre *et al*.^45^ and Laurance *et al*.^46^ showed that topography was one key factor explaining species richness and beta diversity. Thus, the inclusion of terrain attributes into models can be expected to increase the strength of the relationship of soil characteristics to P&CA^47^.

In the framework of the BEF China research group, a multidisciplinary research unit focusing on the relationship between biodiversity and ecosystem services, 27 Comparative Study Plots (CSP, 30 m × 30 m each) were established in a Chinese secondary forest (Gutianshan National Nature Reserve, GNNR) with different stand ages^48^. Within this framework, this study aims to:

(i) detect depth-dependent relationships between soil properties
(ii) quantify the importance of stable and dynamic soil properties on P&CA of tree and herb layer in different soil depths
(iii) determine the critical soil depth columns at which the relationships between soil characteristics and P&CA are at maximum.

## Results

### Depth-dependent relationships between soil properties

**Figure 1:**
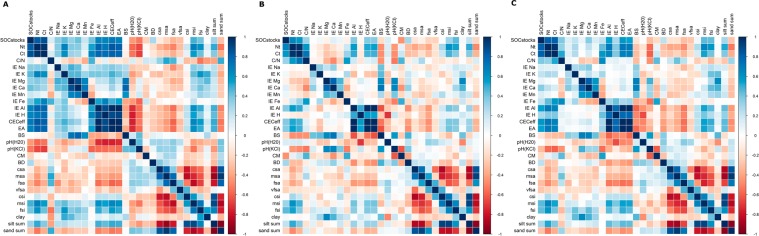
Spearman rank correlations among all measured soil properties (stable and dynamic) of all 27 Comparative Study Plots. Blue and red colors symbolize positive and negative correlations, respectively. (**A**) depth increment 0–20 cm, (**B**) depth increment 20–50 cm and (**C**) depth increment 0–50 cm. Those three soil depth columns are shown as example of all six depth increments investigated.

Soil properties were correlated differently among each other and with soil depth (Fig. 1). In general, stable and dynamic soil properties were correlated strongest among each other at shallow depths (Fig. 1a), whereas these correlations became weaker with increasing depth of the soil horizons from which the soil properties were derived (Fig. 1b,c). In more detail, three cases could be distinguished: (a) dynamic soil properties were more weakly correlated among each other with increasing depth, for instance CEC_eff_ with ion equivalent (IE) Al, SOC_stocks_ with pH (KCl), as well as pH (KCl) with IE Al, (b) dynamic soil properties changed the direction of their covariation, for instance the positive relationship between IE of K, IE of Mg and IE of Ca with CEC_eff_, N_t_ and C_t_, respectively, changed into a negative relationship, (c) dynamic soil properties were positively correlated with each other at shallow depth, but not at deeper depths, for instance correlations between SOC stocks and pH (H_2_O) with CEC_eff_ and IE of Al and H. As a consequence, the effect of soil properties and the strength of their relationship to P&CA_res_ such as biodiversity or biomass changed with respect to soil sampling depth.

### Importance of stable and dynamic soil properties in relation to P&CA_res_

**Figure 2:**
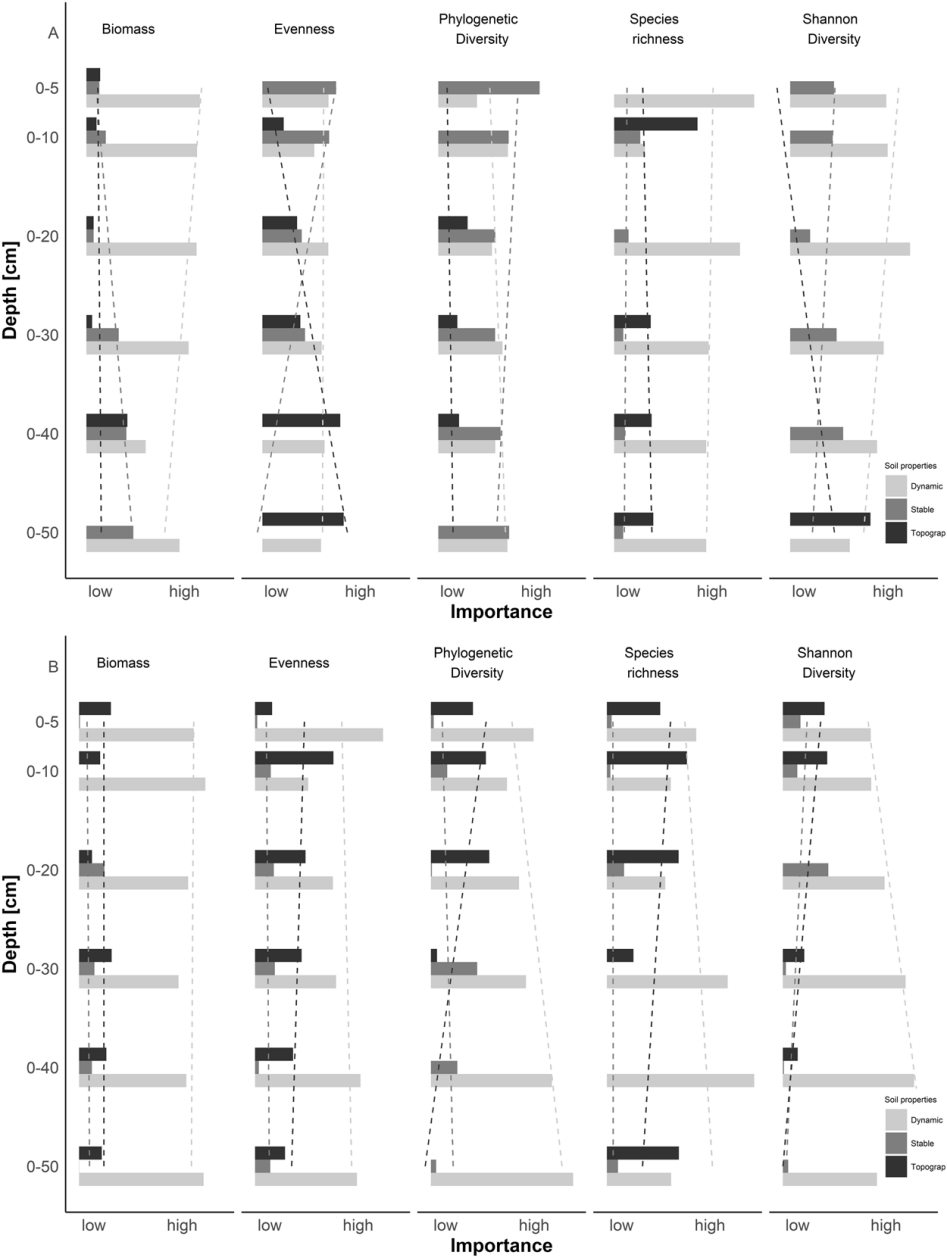
Intra-model variable importance comparison of stable soil properties (grey), dynamic soil properties (light grey) and terrain attributes (dark grey) explaining productivity and community assembly of tree (**A**) and herb layer (**B**) over six soil depth columns. Dashed lines symbolize trends. Variable importance was measured as percentage sum of squares on total sum of squares. Bars do not show total variance of each model.

The importance of stable and dynamic soil properties as well as terrain attributes in the relationship to herb and tree layer P&CA_res_ changed with depth (Fig. 2). Regardless of a specific model, dynamic soil properties lost importance with depth in predicting tree layer P&CA_res_ (Kendall’s tau across all variables: p < 0.05), while the importance of stable properties in predicting tree layer P&CA_res_ remained constant with depth. Considering herb layer P&CA_res_, only the importance of terrain attributes changed with depth (positive effect). Regarding the tree layer (Fig. 2a) and focusing on tree age and biomass, the importance of stable and dynamic properties increased and decreased with depth, respectively. Terrain attributes gained importance with depth in the relationship to Evenness and Shannon index, while for those P&CA_res_, stable soil properties lost importance with depth. Stable and dynamic soil properties did not change importance in the relationship to species richness with depth. Regarding the herb layer (Fig. 2b) and the relationship to biomass and evenness, stable and dynamic soil properties did not change in their importance. However, with depth, dynamic soil properties gained importance focusing on phylogenetic diversity, species richness and Shannon index while terrain attributes lost importance. As importance of different soil properties within a model cannot be directly linked to overall goodness-of-fit of each model, each model’s explained variance needs to be assessed.

### Determination of a critical soil depth to maximize relationship strength between soil properties and P&CA_res_

Importance of tree age as prominent predictor of P&CA_res_ ranged from high influence (adj. R^2^ of 0.72 for biomass) to low influence (adj. R^2^ of 0.04 for species richness) and was significantly higher for tree than for herb layer P&CA_res_ (see Table 3). Since the influence of tree age was high, further results were corrected by that impact.

**Figure 3:**
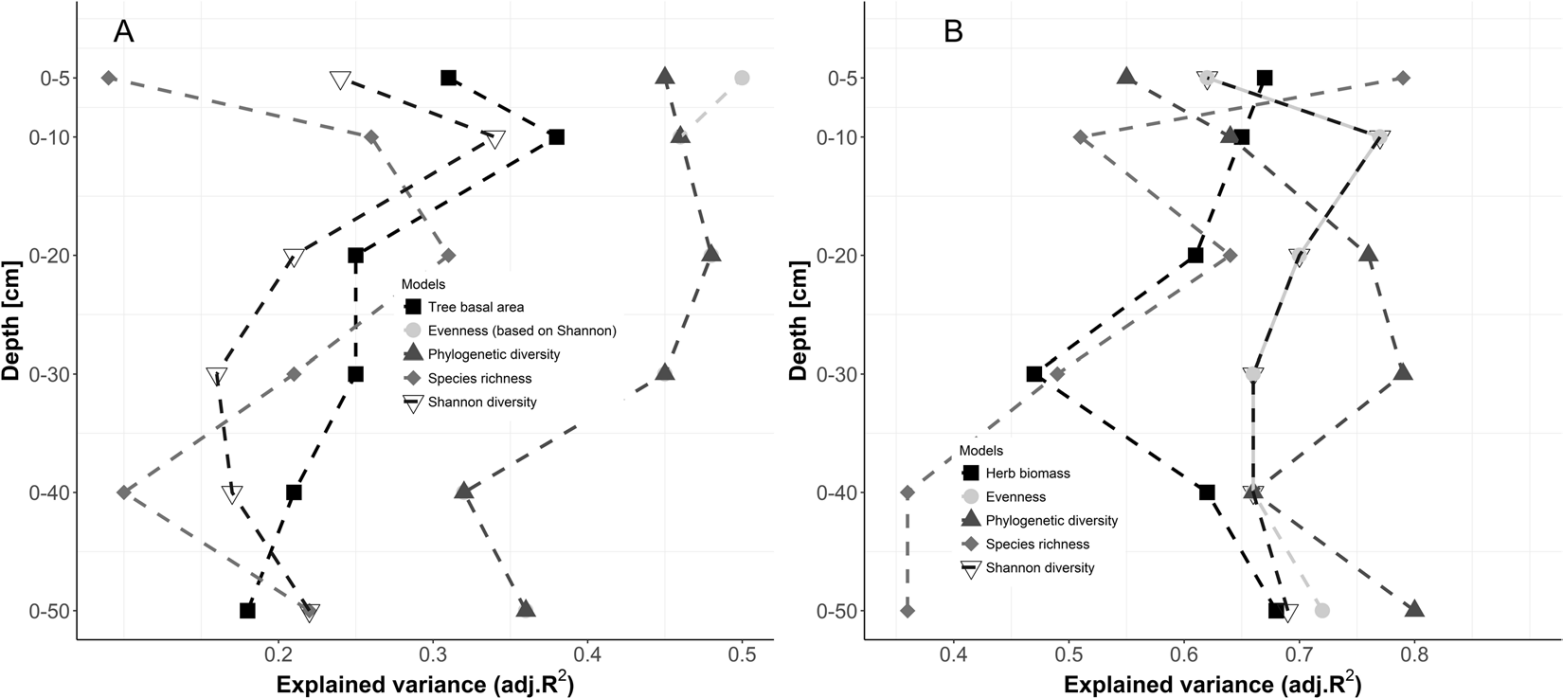
Strength of models (using explained variance) across different soil depth columns explaining productivity (biomass) and community assembly (Shannon diversity, Evenness, Phylogenetic diversity and species richness) of tree (**A**) and herb (**B**) layer. Different colors and symbols represent different models. Dashed lines do not mark a continuity over depth, they help to identify the most suitable depth increment.

Soil properties and terrain attributes explained approximately 64% of the total variance (R^2^) of the herb layer, but only 31% of the tree layer P&CA_res_ (Fig. 3). Strongest relationships (highest R^2^) to tree layer P&CA_res_ were found using soil data from 0–10 cm and 0–20 cm depth, except for species richness and phylogenetic diversity which showed strongest relationship using data from 0–30 cm. Comparing the different P&CA_res_, phylogenetic diversity and evenness showed strong relationships to soil data (R^2^ > 0.5), whereas species richness and Shannon diversity exhibited lower R^2^ values (Fig. 3a). Considering herb layer P&CA_res_, strongest relationships were found using soil data not deeper than 0–30 cm, except for phylogenetic diversity that achieved higher R^2^ values than all other P&CA. In the herb layer, biomass and species richness showed the weakest relation to soil properties and terrain attributes (R^2^ < 0.6, Fig. 3b).

**Figure 4:**
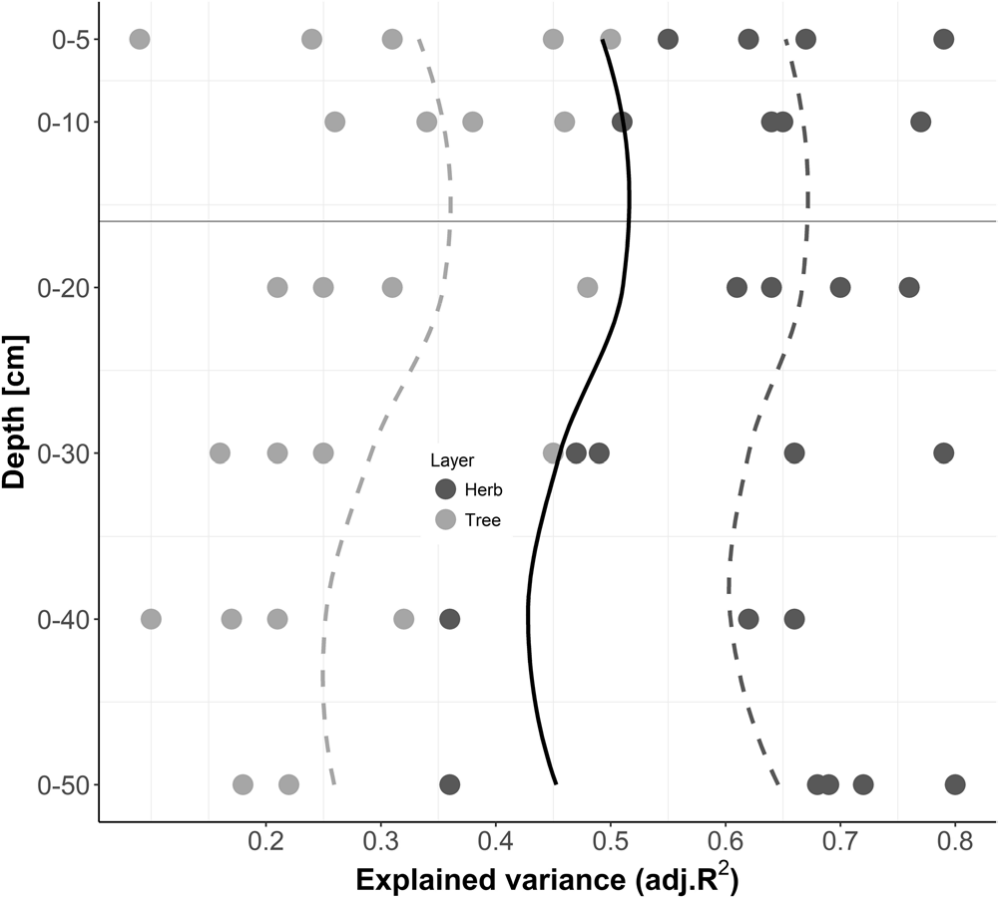
Strength of models (using explained variance) across different soil depth columns explaining productivity (biomass) and community assembly (Shannon diversity, Evenness, Phylogenetic diversity and species richness) of tree (dark dots) and herb (bright dots) layer. The maximum LOWESS fit characterizes the optimum soil depth (dashed dark grey line and dashed light grey line symbolizes fit to tree and herb layer, respectively). Solid black line symbolizes fit across both layers. Solid light grey line shows depth-specific maximum of fit.

There was no common critical soil depth for all response variables. For example, tree basal area was best explained by using soil data of 0–10 cm depth while species richness was best explained with soil data of 0–20 cm (Fig. 3a). However, as the strength of the relationships (measured as variance explained) across all models followed a 3^rd^ order polynomial function (Fig. 4), for this specific study area we propose a critical soil depth of 16 cm. This soil depth column symbolizes the maximum of the fitting function (Fig. 4).

Focusing on specific soil properties in the models, our study showed that CN ratio, coarse-sized sand, pH (H_2_O), bulk density and elevation were the five most prominent variables in relation to P&CA_res_ (Table 4). Differences in the importance of those soil properties occurred between the models of herb and tree layer. While the occurrence of CN ratio in tree layer models was ranked tenth, in herb layer models it took first place. Coarse-sized sand was second prominent in tree layer models, while it was tenth prominent in relation to herb layer P&CA_res_ (Table 4).

## Discussion

### Depth-specific interactions of soil properties

The results showed that soil properties and their relationship among each other are depth-specific for different soil depth columns. This depth-specific behavior can be addressed highlighting three processes in soils: (1) stronger influence of biogenic processes in shallow depths sections as opposed to stronger influence of geogenic processes in deeper depth increments^49^, (2) higher concentration of nutrients due to decreasing amount of pore water with depth, and (3) slower chemical reactions due to lower maximum soil temperatures with depth. These three processes do not only lead to different correlations among soil properties at different depths, but also to different values of soil properties, thus providing differences in resource supply for species to which they may respond with different rooting strategies.

Especially the interplay between stable and dynamic properties was strongly depth-specific, while stable properties did not show large differences in correlation among each other, pointing to inherent conditions throughout the whole soil profile. In accordance to those findings, a study from the Amazon basin revealed that BD can be predicted using dynamic soil properties such as C content and that the accuracy of the prediction is depth-specific^50^. Franzluebbers^51^ showed that the relationship between soil porosity (texture) and SOC was depth-dependent.

In our study, exchangeable K and CEC_eff_ were positively correlated between 0–20 cm while they were negatively correlated in the 20–50 cm depth increment. However, the correlation between exchangeable K and base saturation did not change with depth. The depth-specific CEC_eff_ decrease was mostly influenced by exchangeable Al and only marginally by exchangeable K. In addition, a depth-increasing effect of exchangeable Al pointed to high influence on CEC_eff_ even with depth, whereas exchangeable K must be limited in deeper depths leading to negative correlation to CEC_eff_ in the 20–50 cm depth increment. It is assumed that exchangeable K is dominantly used by plants in deeper soil depths rather than exchangeable Al, hence explaining the increase of CEC_eff_ combined with a decrease of exchangeable K with depth. Many nutrients were found to be enriched in the topsoils, which can be interpreted as an effect of more intense decomposition and a dilution effect of K through nutrient uptake with increasing depth.

Weaker correlations among dynamic soil properties (e.g. CEC_eff_ and exchangeable Al or exchangeable H, and SOC_stocks_ and pH (KCl), and pH (KCl) and exchangeable Al) can be explained by a dilution effect which can favor the transport of specific ions in depth. In this study area, Cambisols with high buffer capacity may disfavor transport of Al and H only slightly compared to K and Na due to pH variation changing correlations of K and Na. If less Al is present in deeper soil, other ions may take its part hence weakening the correlations among Al and other dynamic soil properties such as CEC_eff_ and pH (KCl). Texture (e.g. fractions of medium-sized sand and silt) was considered a stable soil property and thus did not vary much with depth and, in addition, with time. It was mainly influenced by parent material, which remains constant throughout the soil profile.

### Importance of stable and dynamic soil properties in relation to P&CA_res_

Stable and dynamic soil properties as well as terrain attributes showed differences in predicting P&CA_res_ at different depths. Considering herb and tree layer models independent of soil depth, dynamic soil properties were more important than stable soil properties and terrain attributes, emphasizing the dynamic interaction between soils and plants^22^. Nonetheless, both stable and dynamic soil properties outplayed terrain attributes.

Terrain attributes significantly gained importance with depth and were more important in the relationships to herb layer than to tree layer P&CA_res_. This layer might be more dependent to limitations in sunlight and water availability compared to the tree layer (e.g. where less shading effects occur). It has been shown that especially aspect determines the available sun light^52^ and thus drives photosynthesis activity while slope and to a lesser extent also elevation affect water availability^53^. As revealed by another study in the GNNR, slope was found to affect tree morphology^54^ as well as biomass and biodiversity. In addition, elevation and terrain convexity were found to affect species richness and composition in the GNNR^45^. In central Amazonia, less steep areas had higher nutrient soils and provided a greater tree diversity^46^. In general, the influence of terrain attributes on P&CA_res_ should not be neglected as shown by their presence in all models relating soil properties and terrain attributes to herb and tree layer P&C_res_A. However, tree age was only marginally influenced by terrain attributes, which shows that past logging activities were not concentrated in specific landscape types^48^.

In contrast, stable soil properties were of higher importance to tree layer P&CA_res_ compared to herb layer P&CA_res_, which probably is explained by a more confined rooting space to the surface horizons^55,56^. In addition, stable soil properties, which are influenced by the parent material, gained importance with depth. This was especially true for tree basal area, being a proxy for tree growth rather than tree diversity. The relationship between stable soil and terrain attributes may be caused by the fact that stable soil properties served as a proxy for relief, thus explaining variance contributed also by terrain attributes.

In the tree layer, the importance of dynamic soil properties decreased with depth for half of the cases. The deeper the soil depth increment considered, the clearer a dilution effect became visible for dynamic soil properties, thus weakening the interplay between P&CA_res_ and soils. However, herb layer biomass shows the same behavior to dynamic soil properties across different depths.

### Relationship strength of soil properties and terrain attributes to P&CA leads to a critical soil depth

Models obtained adj. R^2^ > 0.20 for all P&CA_res_ (expect species richness of the tree layer). The strongest relationships were found for phylogenetic diversity and species richness of the herb layer with adj. R^2^ = 0.81. Thus, soil properties and terrain attributes are highly valuable predictors of P&CA_res_ (also see^5,33,46^). This finding is supported by the fact that results were obtained after the exclusion of tree age effects on P&CA_res_. In particular, tree layer P&CA_res_ were affected by tree age. In contrast, herb layer P&CA_res_ showed almost no influence of tree age. Thus, tree age is driving large differences in tree layer P&CA_res_ variables among plots and decreases explicability of solely soil properties. Nevertheless, maximum predictability, e.g. considering plant growth, was found in specific depths hence explaining depth-specific variations in the strength of the relationship to herb and tree layer. These results outperformed findings of other studies with high explained variances and emphasizes the general suitability of the approach incorporating soil properties, in particular of different depths, and terrain attributes^35,46,57^.

Variations in relationship strength were higher for the tree than for the herb layer P&CA_res_. Higher impact of single species and a more heterogeneous pattern in low mixture stands based on lower biodiversity indices in the tree than in the herb layer might lead to the larger prediction variations for the tree layer. It is known that high-diversity stands may take up nutrients more efficiently in all soil depths compared to low-diversity stands^58^. In addition, high-diversity stands tend to have increased fine-root growth compared to monoculture stands^59^.

In addition, different rooting strategies of the tree layer compared to the herb layer explain lower prediction accuracies for the tree layer. It was shown that shallow roots (probably related to herb layer) can have competitive advantages over deeper roots^60^. In addition, root competition affects plant species diversity at the community level and primary production at the ecosystem level^60^. However, maximum rooting depth of both herbs and trees by far exceed soil depths (max. < 70 cm) in the study area^61^. It is also known that herb layer influences tree layer, in particular tree layer seedlings and saplings, through a “bottom-up” effect by filtering tree regeneration through herb stratum and competition of nutrients^62,63^. This might be one reason for the stronger relationship of soil properties and terrain attributes to herb layer P&CA_res_. However, there may also be a “top-down” effect as tree layer regulates light transmittance into the herb layer^64,65^. The similarities in rooting depth and the compensation of a “bottom-up” with a “top-down” approach were well represented in the relationship strength of tree and herb layer models.

The study’s results clearly showed that there were specific soil depth columns that were superior in explaining specific P&CA_res_, i.e. species richness of herb layer was best explained using soil data of 0–5 cm while biomass of herb layer was best explained using soil data of 0–50 cm. However, most soil properties and terrain attributes investigated in this subtropical forest ecosystem showed maximum correlations to most P&CA_res_ characteristics at 0–10 cm and 0–20 cm (maximum of fitting function for herb and tree layer), with a similar fitting curve pattern. Hence, a critical soil depth column of 0–16 cm is suggested. As both critical soil depths for the herb and the tree layer are the same, a strong interaction between the two layers can be suggested (c.f.^57^). We assume that within the first 16 cm of a soil, a stronger interrelationship between stable and dynamic soil properties influences P&CA_res_ most, thus leading to strongest relationships. If the different behavior of soil properties with depth is not considered and only averaged results across the whole soil profile are used, a dilution effect (equal to weaker relations) may be noticed due to changes in the effect of and correlation among soil properties. Nevertheless, phylogenetic diversity and biomass of the herb layer were better predicted using broader soil depth increments. These results point out that critical soil depths are regional- and variable-specific.

## Summary and Conclusion

This study analyzed depth-specific soil – plant relationships using data of 27 CSPs in a subtropical forest in SE China. Models were built investigating the relationship of 17 stable and dynamic soil properties as well as terrain attributes to P&CA. This study defines a “critical soil depth” as being the lower boundary of the sampling extent necessary to gain optimum results for analyzing relationships of soils to P&CA in a particular area. We showed that

(i) soil properties behave differently with depth and
(ii) the interplay between stable and dynamic soil properties as well as terrain attributes is important for the prediction of P&CA.
(iii) for specific P&CA characteristics, there is a specific critical soil depth in which the interplay between those properties is ideal.

In the investigated subtropical forest, a soil depth column of 0–16 cm was best explaining most of the P&CA characteristics. This column can be sampled as one bulk sample or as different subsamples, which then have to be bulked. The approach of this study achieved higher prediction strength than most other studies thereby pointing to a superior description of relationships by sampling the appropriate soil data (after correction for stand age). However, interactions between soils and plants through stand age and long-lasting mass transfer might shift the critical soil depth with time. Soils different from Cambisols found at this particular study site may have different properties. Thus, the interplay between stable and dynamic properties might take place in different depth increments also resulting in a different critical soil depth. In addition, overall soil depth and depth of the organic layer can influence the critical soil depth. Our data showed that it is not sufficient to solely sample topsoil or to use average data across the available depth of soils. If the whole soil profile is considered, a dilution effect can occur leading to lower strength in the relationships of soil properties to P&CA. The strength of the relationship between soil properties and P&CA is constrained by the variables chosen. The intrinsic critical soil depth should be considered stand-specific and calls for re-evaluation in different areas and habitats. Further research in different ecosystems and other parts of the world are also required to produce a more general model.

## Material and Methods

### Study site

The study was conducted in the Gutianshan National Nature Reserve (GNNR), Zhejiang Province, P.R. of China (N 29° 14.657′ and E 118° 06.805′). The GNNR covers an area of around 81 km^2^. The topography is characterized by steep to very steep slopes (14° to 47° with mean 33°) at elevations from 251–903 m with a mean of 547 m a.s.l. The climate at the GNNR is typical of subtropical summer monsoon regions with a mean annual temperature of 15.3 °C and mean annual rainfall of 1964 mm^66^. The soils are dominated by Cambisols, derivates of Cambisols and partly colluvial deposits and Luvisols^67^ developed in weathering material from granite and gneiss^66^. However, heterogeneity of parent material throughout the study area is small compared to heterogeneity of relief. Thus, only relief parameters enter the subsequent analyses.

Within the mixed broad-leaved forest of the GNNR, the BEF China project^68^ established 27 randomly selected Comparative Study Plots (CSPs), each of 900 m^2^ in size (30 m × 30 m) with varying biomass and tree and herb biodiversity^48^. The CSPs cover a successional gradient from 20 to more than 115 years. Dominant tree species are *Castanopsis eyrei* and *Schima superba*^69^.

### Soil sampling & laboratory analyses

In the study area, soils were shallow (average soil depth: 67 cm)^70^. Soil samples were taken in the center of nine regular subplots within each of the 27 CSPs in 2009. Six depth intervals (0–5 cm, 5–10 cm, 10–20 cm, 20–30 cm, 30–40 cm, 40–50 cm) were sampled at every subplot and then pooled per depth interval, resulting in 162 samples (27 CSPs × six depth increments). The depth intervals were chosen in the broader context of the BEF China project, which aims to analyze the effect and importance of different biodiversity variables. For each sample, 29 soil characteristics were analyzed (see Table 1 for the example of consolidated data from 0–50 cm, data of all other depth columns can be found in Supplement 1), classified into stable and dynamic soil properties. As the geological timeframe for this study is centuries (oldest stand > 80 years) and management intensity was low or absent in the last decades, we considered bulk density (BD) as stable property, whereas, for instance, base cations and effective cation exchange capacity (CECeff) were considered dynamic properties because they were easily available to plants.

**Table 1:** Laboratory analysis data for all 27 CSPs for a soil depth column of 0 cm to 50 cm (calculated as weighted mean of six sampled depth intervals).

Description	Abbrev.	Min	SD	Median	Mean	Max
**Dynamic soil properties**						
pH H_2_O	pH_H20_	4.32	0.17	4.70	4.70	5.06
pH KCl	pH_KCl_	3.68	0.10	3.81	3.83	4.01
Effective cation exchange capacity	CECeff	28.61	14.46	45.81	47.97	82.28
Na [µmol_c_ g^−1^]	IE Na	0.10	0.13	0.19	0.24	0.29
K [µmol_c_ g^−1^]	IE K	0.73	0.29	1.19	1.21	1.83
Mg [µmol_c_ g^−1^]	IE Mg	0.36	0.60	1.00	1.17	3.50
Ca [µmol_c_ g^−1^]	IE Ca	0.45	1.28	1.64	2.01	6.08
Mn [µmol_c_ g^−1^]	IE Mn	0.09	0.33	0.36	0.49	1.44
Fe [µmol_c_ g^−1^]	IE Fe	0.09	0.22	0.29	0.31	1.16
Al [µmol_c_ g^−1^]	IE Al	22.64	13.71	37.46	41.04	74.64
H [µmol_c_ g^−1^]	IE H	0.73	0.54	1.43	1.53	2.88
Base saturation [%]	BS	4.06	3.91	8.74	9.50	21.07
Exchangeable Acidity [%]	EA	23.90	14.18	38.70	43.37	78.54
Total organic nitrogen [mass-%]	N_t_	0.045	0.04	0.08	0.09	0.22
Total organic carbon [mass-%]	C_t_	0.71	0.76	1.21	1.43	4.18
Soil organic carbon stocks [t ha^−1^]	SOCstocks	0.23	0.49	0.78	0.90	2.52
C:N ratio	CN	12.77	1.74	14.75	15.12	18.92
**Stable soil properties**						
Bulk Density [g cm^−3^]	BD	0.81	0.15	1.08	1.08	1.46
Coarse Material [%]	CM	0	18.51	15.82	20.12	72.60
Coarse-sized sand [%]	csa	4.06	6.47	15.13	15.65	29.72
Medium-sized sand [%]	msa	3.60	4.28	14.12	13.12	20.78
Fine-sized sand [%]	fsa	1.55	1.46	5.29	5.15	8.37
Very fine-sized sand [%]	vfsa	4.59	2.65	9.22	8.71	13.43
Coarse-sized silt [%]	csi	5.63	5.93	13.28	15.21	26.27
Medium-sized silt [%]	msi	8.79	3.86	11.59	13.02	24.64
Fine-sized silt [%]	fsi	5.28	2.01	7.68	7.95	13.69
Clay [%]	clay	11.70	3.35	21.70	21.21	26.40
Silt sum [%]	si_sum_	22.60	10.04	33.70	36.17	64.40
Sand sum [%]	sa_sum_	13.90	10.38	43.80	42.58	60.00

All elements are exchangeable elements and are shown in ion equivalents. Soil texture classes are shown according to^72^. Abbrev.: abbreviation, Min: minimum, SD: standard deviation, Max: maximum, IE: ion equivalent. Information on data of all other soil depth columns can be found at Supplement Tables 1–6.

Sample preparation was done by hand sorting of coarse plant and animal residuals, sieving (< 2 mm) and grinding of air-dried soil samples. Soil pH and concentration of H were measured potentiometrically in both 1 M KCl and bi-distilled H_2_O (WTW pH-meter with Sentix81 electrodes, Weilheim, Germany). Before ICP-OES measurements, the soil samples were percolated with an unbuffered 1 M NH_4_Cl solution (CEC_eff_) to evaluate the overall assessment of the potential fertility of the soil. Effective cation exchange capacity (CEC_eff_), exchangeable acidity (EA, using Al and H ions) and ion equivalents (IE) of Na, K, Mg, Ca, Mn, Fe as well as Al were measured with ICP-OES (Perkin Elmer Optima 5300 DV ICP OES Waltham, MA, USA). Base saturation percentage (BS) was calculated as proportion of the CEC_eff_ accounted for by exchangeable bases Na, K, Mg and Ca. Total organic carbon (C_t_) and total organic nitrogen (N_t_) were measured using heat combustion (VARIO EL III, Elementar, Hanau, Germany). Given the acidic soil conditions of the CSPs, inorganic carbon (C) does not occur and C_t_ represents the soil organic carbon content (SOC). SOC stocks (t ha^−1^) to a depth of 50 cm were calculated according to^71^:

1 $$SO{C}_{stocks}=\sum _{i=1}^{n}\,(Dept{h}_{i}\times (\frac{Ct}{10})\times (\frac{BD}{1000})\times (1-(CM/100)))/100$$

where Depth_i_ is a specific depth increment (m), SOC (g C 100 g^−1^ = mass-%) represents SOC related to the increment, BD (g cm^−3^) is the bulk density recalculated as weighted mean, and CM (%) is the fraction of coarse material, estimated following the German guidelines for soil description^72^. Bulk soil density (BD) was determined gravimetrically on volumetric samples. Particle size analysis was done by a combined pipette and sieving method (seven fractions, Koehn, DIN 19683-1). CSP means of those soil properties were used for further data analyses.

### P&CA: biomass, biodiversity indices and tree age

To investigate soil - plant relationships, we analyzed the herb layer (≤1 m height) separately from the tree layer (>1 m height). We used characteristics which have shown to be relevant in previous analysis^18,57,73^. For each layer we used species richness, Shannon index and evenness based on the Shannon index as descriptors for taxonomic diversity. We also used phylogenetic diversity which was found to be a strong predictor of ecosystem processes^74^.

Species richness, Shannon index, Evenness based on Shannon index and Phylogenetic diversity were used to describe attributes of tree and herb, layers (Table 2). In addition, biomass estimated by basal area and total biomass were used for the tree and herb layer, respectively. Species richness was defined as number of tree and shrub species exceeding 1 m height. Shannon index is given by

2 $$H=-\,\sum {p}_{i}\times \,\mathrm{log}({p}_{i})$$

with p_i_ being the relative abundance of the i^th^ species^75^. Evenness based on Shannon index is given by $E=\frac{H}{\mathrm{log}({\rm{species}}\,{\rm{richness}})}$. The phylogeny was calculated based on sequence information (matK, rbcL, and ITS region) for all species, or their closest relatives, from GenBank or de novo using standard barcoding protocols. A maximum-likelihood tree was computed and dated using nonparametric rate smoothing and using published fossils as age constraints^74^. To avoid potential bias in the analysis of phylogenetic patterns due to their disproportionately long-branch lengths, non-angiosperm and the only bamboo species, which generally occurred at low frequencies within the study area, were excluded from calculating phylogenetic diversity. Tree age was determined using stem cores of the fifth largest tree individual in each CSP (combined measurement of diameter at breast height and tree ring width). Basal area was calculated using the formula of a circle of the corresponding diameter at breast height (DBH) of all trees exceeding 10 cm DBH of each CSP^76^. Herb layer biomass was determined as complete oven-dried harvest of all herb layer aboveground biomass in the centered 100 m^2^ of each CSP^57^. All P&CA attributes were measured as a single measurement in time in 2008 and are representative of the complete CSP.

**Table 2:** Descriptive statistics of four biodiversity indices, biomass/basal area and tree age of 27 plots.

	Aboveground P&CA signals	Min	SD	Median	Mean	Max
TL	Shannon index	1.80	0.42	2.85	2.79	3.41
	Species richness	25.00	10.34	39.00	41.81	69.0
	Evenness	0.53	0.09	0.77	0.75	0.85
	Phylogenetic diversity	2.01	0.17	2.63	2.58	2.77
	Basal area [m^2^/plot]	0.22	1.29	2.07	2.15	4.93
	Tree age [a]	21.72	26.05	71.71	67.40	115.54
HL	Shannon index	1.59	0.60	3.49	3.25	3.90
	Species richness	25.00	10.43	42.00	42.89	71.00
	Evenness	0.48	0.15	0.94	0.87	1.00
	Phylogenetic diversity	2.03	0.46	3.76	3.50	3.84
	Total biomass [g m^−2^]	0.19	17.52	17.92	21.08	74.60

P&CA: productivity and community assembly, TL: tree layer, HL: herb layer, Min: minimum, SD: standard deviation, Max: maximum.

**Table 3:** Assessing influence of tree age in predicting biomass, Shannon diversity, Evenness, Species richness and Phylogenetic diversity of tree and herb layer.

Model	Adjusted. R^2^ of tree layer	Adjusted. R^2^ of herb layer
Basal area (biomass) ~ log(Tree age)	0.72	0.04
Phylogenetic diversity ~ log(Tree age)	0.35	<0.01
Evenness ~ log(Tree age)	0.45	<0.01
Shannon ~ log(Tree age)	0.35	<0.01
Species richnnes ~ log(Tree age)	0.04	0.03

**Table 4:** Occurrence (count) of the ten most prominent soil properties and terrain attributes in all models, tree layer models and herb layer models.

Soil property/terrain attribute	Total occurrence	Occurrence in tree layer models	Occurrence in herb layer
C:N	30 (1)	2 (10)	28 (1)
Coarse-sized sand	27 (2)	18 (2)	9 (7)
pH (H_2_O)	25 (3)	20 (1)	5 (11)
Bulk density	21 (4)	9 (3)	12 (4)
Elevation	19 (5)	5 (5)	14 (2)
Carbon stocks	15 (6)	4 (8)	11 (5)
Cation exchange capacity (effective)	14 (7)	—	14 (2)
Clay	13 (8)	5 (5)	8 (8)
Fine-sized sand	11 (9)	1 (11)	10 (6)
Slope	11 (10)	4 (8)	7 (10)
Ion equivalent Iron	10 (11)	7 (4)	3 (12)
Very fine-sized sand	9 (12)	1 (11)	8 (8)
Ion equivalent Potassium	8 (13)	5 (5)	3 (12)

Models were used to relate soil properties and terrain attributes to biomass, Shannon diversity, Evenness, Species richness, Phylogenetic diversity and tree age. The rank is displayed in brackets.

### Site characteristics

We included slope, aspect (northness and eastness) and elevation of each CSP as terrain attributes into our analyses (Supplement Table 7).

### Data analyses

Soil properties were aggregated into distinct soil depth columns (0–5 cm, 0–10 cm, 0–20 cm, 0–30 cm, 0–40 cm and 0–50 cm) to model P&CA in order to find the critical soil depth in which relationships are strongest. The depth-specific aggregation was accomplished using depth-interval-weighted means of each soil property (i.e. to yield soil data of 0–20 cm interval, data from 0–5 cm and 0–10 cm entered the calculation as one quarter each and the interval of 10–20 cm entered the calculation as two quarters), except for SOC stocks which were cumulated to the total absolute amount of SOC stocks within a distinct depth.

N_t_, C_t_ (here: SOC stocks were used instead), IE of Ca, Mn, Al and H, EA, medium-sized sand, coarse silt, medium-sized silt, total silt and total sand were excluded prior to analyses due to multicollinearity (Spearman’s r >0.7). Thus, models were built using 17 independent variables on each of four biodiversity indices and biomass (basal area for tree layer) as dependent variables (Table 4). In addition, each model was corrected for the influence of tree age (log-linear) using linear models and all following analysis were done using residuals of these corrected linear models (see Table 1). The following term P&CA_res_ refers to P&CA after correction for tree age.

In total, 60 final linear models were derived (6 depths × 10 P&CA_res_ (5 × herb layer + 5 × tree layer)). To identify the best set of independent variables for each of those models, automated model selection was done using the generic algorithms of the R package “glmulti”^77^ in which the intercept and main effects were considered. Akaike corrected Information Criterion (AICc) was used to find the best model since AICc outranks AIC for small sample sizes (n = 27). However, AICc was not suitable (only intercept models were generated) for species richness models and thus, in this case, models were selected based on AIC. Best models with the appropriate candidate set were refit and assessed by their adjusted R^2^ and the overall significance of the model. Model residuals did not show any violation of model assumptions (normality and homogeneity of variances).

In a further step, R^2^ values of each model for increasing depth increment were fitted using locally weighted scatterplot smoothing (LOWESS) with a parameter set of degree = 2 and span = 1. Parameters were set according to lowest residual standard error of the models (combination of degree 1 and 2 was tested with span of 0.75,1 and 2) and by-eye assessing of overfitting problems (due to similar errors of different parameter settings). This LOWESS function was applied to tree, herb and the mean of both vegetation layers. The maximum of this function was determined as critical soil depth in which strongest relationships between soil and P&CA_res_ can be found.

The importance of stable and dynamic soil properties as well as terrain attributes in the relationship to P&CA_res_ in different soil depths was assessed using ANOVAs sum of squares of each independent variable as percentage of total sum of squares of all independent variables in each model. Kendall’s tau was used to assess differences in importance of soil properties and terrain attributes with depth. Spearman’s r was used to assess different relationships among soil properties in different depths.

All analyses were carried out using R v.2.15.3^78^ and its incorporated “stats” package.

## Supplementary information

Supplementary Dataset 1

## Data Availability

The datasets generated within this study result from the BEF China experiment and are either available in the supplement or from the BEF China data portal (URL: http://china.befdata.biow.uni-leipzig.de/) and upon request from the corresponding author.
