# Supplementary material for: The strength of soil-plant interactions under forest is related to a Critical Soil Depth
Source: Sci Rep. 2019 Jun 14;9:8635. doi: 10.1038/s41598-019-45156-5 (PMC6572823; doi:10.1038/s41598-019-45156-5)
Supplement: Supplementary file 1 — Supplementary Dataset 1 [file 41598_2019_45156_MOESM1_ESM.docx]

**Supplement and supplement information**

**The strength of soil-plant interactions under forest is related to a Critical Soil Depth**

Philipp Goebes^1^, Karsten Schmidt^1^, Steffen Seitz^1^, Sabine Both^2,3^, Helge Bruelheide^2^, Alexandra Erfmeier,^4^ Thomas Scholten^1^ and Peter Kühn^1^

1 Institute of Geography, Soil Science and Geomorphology, University of Tübingen, Rümelinstrasse 19-23, Tübingen, Germany

2 Institute of Biology, Geobotany and Botanical Garden, Martin-Luther University Halle-Wittenberg, Am Kirchtor 1, Halle, Germany

3 Environmental and Rural Science, University of New England, Armidale NSW, 2351, Australia

4 Institute of Ecosystem Research, Kiel University, Olshausenstrasse 75, Kiel, Germany

**Supplement. Table 1.** Laboratory analysis data for all 27 CSPs and 0-5 cm soil depth columns. All elements are exchangeable elements and are shown in ion equivalents. Soil texture classes are shown according to ^71^. Data of specific soil columns were calculated as weighted mean of sampled depth intervals (except for soil organic carbon stocks). Abbrev.: abbreviation, Min: minimum, SD: standard deviation, Max: maximum, IE: ion equivalent.

| Description | Abbrev. | Min | SD | | Median | | Mean | | Max | |  |
| --- | --- | --- | --- | --- | --- | --- | --- | --- | --- | --- | --- |
| Dynamic soil properties |  |  | |  | |  | |  | |  | |
| pH H_2_O | pH_H20_ | 4.32 | | 0.17 | | 4.70 | | 4.70 | | 5.06 | |
| pH KCl | pH_KCl_ | 3.68 | | 0.10 | | 3.81 | | 3.83 | | 4.01 | |
| Effective cation exchange capacity | CECeff | 28.61 | | 14.46 | | 45.81 | | 47.97 | | 82.28 | |
| Na [µmol_c_ g^-1^] | IE Na | 0.10 | | 0.13 | | 0.19 | | 0.24 | | 0.29 | |
| K [µmol_c_ g^-1^] | IE K | 0.73 | | 0.29 | | 1.19 | | 1.21 | | 1.83 | |
| Mg [µmol_c_ g^-1^] | IE Mg | 0.36 | | 0.60 | | 1.00 | | 1.17 | | 3.50 | |
| Ca [µmol_c_ g^-1^] | IE Ca | 0.45 | | 1.28 | | 1.64 | | 2.01 | | 6.08 | |
| Mn [µmol_c_ g^-1^] | IE Mn | 0.09 | | 0.33 | | 0.36 | | 0.49 | | 1.44 | |
| Fe [µmol_c_ g^-1^] | IE Fe | 0.09 | | 0.22 | | 0.29 | | 0.31 | | 1.16 | |
| Al [µmol_c_ g^-1^] | IE Al | 22.64 | | 13.71 | | 37.46 | | 41.04 | | 74.64 | |
| H [µmol_c_ g^-1^] | IE H | 0.73 | | 0.54 | | 1.43 | | 1.53 | | 2.88 | |
| Base saturation [%] | BS | 4.06 | | 3.91 | | 8.74 | | 9.50 | | 21.07 | |
| Exchangeable Acidity [%] | EA | 23.90 | | 14.18 | | 38.70 | | 43.37 | | 78.54 | |
| Total organic nitrogen [mass-%] | N_t_ | 0.12 | | 0.04 | | 0.22 | | 0.24 | | 0.59 | |
| Total organic carbon [mass-%] | C_t_ | 2.33 | | 0.76 | | 4.13 | | 4.35 | | 11.81 | |
| Soil organic carbon stocks [t ha^-1^] | SOCstocks | 0.23 | | 0.49 | | 0.78 | | 0.90 | | 2.52 | |
| CN ratio | CN | 13.60 | | 1.74 | | 18.80 | | 15.12 | | 22.50 | |
|  |  |  | |  | |  | |  | |  | |
| Stable soil properties |  |  | |  | |  | |  | |  | |
| Bulk Density [g cm^-3^] | BD | 0.42 | | 0.15 | | 0.92 | | 0.87 | | 1.46 | |
| Coarse Material [%] | CM | 0 | | 18.51 | | 5.50 | | 8.80 | | 25.00 | |
| Coarse-sized sand [%] | csa | 4.06 | | 6.47 | | 15.13 | | 15.65 | | 29.72 | |
| Medium-sized sand [%] | msa | 3.60 | | 4.28 | | 14.12 | | 13.12 | | 20.78 | |
| Fine-sized sand [%] | fsa | 1.55 | | 1.46 | | 5.29 | | 5.15 | | 8.37 | |
| Very fine-sized sand [%] | vfsa | 4.59 | | 2.65 | | 9.22 | | 8.71 | | 13.43 | |
| Coarse-sized silt [%] | csi | 5.63 | | 5.93 | | 13.28 | | 15.21 | | 26.27 | |
| Medium-sized silt [%] | msi | 8.79 | | 3.86 | | 11.59 | | 13.02 | | 24.64 | |
| Fine-sized silt [%] | fsi | 5.28 | | 2.01 | | 7.68 | | 7.95 | | 13.69 | |
| Clay [%] | clay | 11.70 | | 3.35 | | 21.70 | | 21.21 | | 26.40 | |
| Silt sum [%] | si_sum_ | 22.60 | | 10.04 | | 33.70 | | 36.17 | | 64.40 | |
| Sand sum [%] | sa_sum_ | 13.90 | | 10.38 | | 43.80 | | 42.58 | | 60.00 | |

**Supplement. Table 2.** Laboratory analysis data for all 27 CSPs and 0-10 cm soil depth columns. All elements are exchangeable elements and are shown in ion equivalents. Soil texture classes are shown according to ^71^. Data of specific soil columns were calculated as weighted mean of sampled depth intervals (except for soil organic carbon stocks). Abbrev.: abbreviation, Min: minimum, SD: standard deviation, Max: maximum, IE: ion equivalent.

| Description | Abbrev. | Min | SD | | Median | | Mean | | Max | |  |
| --- | --- | --- | --- | --- | --- | --- | --- | --- | --- | --- | --- |
| Dynamic soil properties |  |  | |  | |  | |  | |  | |
| pH H_2_O | pH_H20_ | 4.00 | | 0.23 | | 4.65 | | 4.61 | | 5.05 | |
| pH KCl | pH_KCl_ | 3.40 | | 0.14 | | 3.75 | | 3.76 | | 4.00 | |
| Effective cation exchange capacity | CECeff | 32.65 | | 24.80 | | 58.50 | | 63.96 | | 142.90 | |
| Na [µmol_c_ g^-1^] | IE Na | 0.10 | | 0.12 | | 0.21 | | 0.24 | | 0.55 | |
| K [µmol_c_ g^-1^] | IE K | 0.90 | | 0.56 | | 1.49 | | 1.60 | | 2.78 | |
| Mg [µmol_c_ g^-1^] | IE Mg | 0.59 | | 1.11 | | 1.79 | | 1.91 | | 5.30 | |
| Ca [µmol_c_ g^-1^] | IE Ca | 0.62 | | 4.22 | | 4.66 | | 5.57 | | 15.70 | |
| Mn [µmol_c_ g^-1^] | IE Mn | 0.18 | | 0.81 | | 1.22 | | 1.25 | | 3.02 | |
| Fe [µmol_c_ g^-1^] | IE Fe | 0.13 | | 0.61 | | 0.70 | | 0.70 | | 3.35 | |
| Al [µmol_c_ g^-1^] | IE Al | 22.13 | | 21.44 | | 44.62 | | 50.42 | | 123.30 | |
| H [µmol_c_ g^-1^] | IE H | 1.10 | | 1.27 | | 2.15 | | 2.30 | | 6.75 | |
| Base saturation [%] | BS | 4.35 | | 7.15 | | 12.10 | | 14.14 | | 33.25 | |
| Exchangeable Acidity [%] | EA | 24.40 | | 22.99 | | 48.50 | | 54.67 | | 133.60 | |
| Total organic nitrogen [mass-%] | N_t_ | 0.10 | | 0.10 | | 0.17 | | 0.19 | | 0.50 | |
| Total organic carbon [mass-%] | C_t_ | 1.78 | | 1.76 | | 3.08 | | 3.42 | | 9.89 | |
| Soil organic carbon stocks [t ha^-1^] | SOCstocks | 1.02 | | 1.69 | | 2.20 | | 2.79 | | 7.62 | |
| CN ratio | CN | 13.55 | | 1.79 | | 18.25 | | 18.02 | | 21.60 | |
|  |  |  | |  | |  | |  | |  | |
| Stable soil properties |  |  | |  | |  | |  | |  | |
| Bulk Density [g cm^-3^] | BD | 0.42 | | 0.20 | | 0.93 | | 0.92 | | 1.46 | |
| Coarse Material [%] | CM | 0.00 | | 9.23 | | 2.25 | | 6.92 | | 35.00 | |
| Coarse-sized sand [%] | csa | 4.15 | | 6.32 | | 13.60 | | 15.01 | | 29.80 | |
| Medium-sized sand [%] | msa | 3.30 | | 4.73 | | 13.95 | | 13.15 | | 20.50 | |
| Fine-sized sand [%] | fsa | 1.35 | | 1.59 | | 5.60 | | 5.31 | | 7.80 | |
| Very fine-sized sand [%] | vfsa | 4.40 | | 2.76 | | 9.80 | | 9.29 | | 14.65 | |
| Coarse-sized silt [%] | csi | 7.95 | | 6.07 | | 14.55 | | 15.97 | | 26.95 | |
| Medium-sized silt [%] | msi | 8.25 | | 3.58 | | 12.35 | | 12.97 | | 23.20 | |
| Fine-sized silt [%] | fsi | 5.35 | | 2.08 | | 7.60 | | 8.10 | | 14.75 | |
| Clay [%] | clay | 10.50 | | 3.58 | | 20.50 | | 20.22 | | 26.00 | |
| Silt sum [%] | si_sum_ | 24.50 | | 9.76 | | 35.00 | | 37.00 | | 63.00 | |
| Sand sum [%] | sa_sum_ | 13.50 | | 11.39 | | 44.00 | | 42.69 | | 57.00 | |

**Supplement. Table 3.** Laboratory analysis data for all 27 CSPs and 0-20 cm soil depth columns. All elements are exchangeable elements and are shown in ion equivalents. Soil texture classes are shown according to ^71^. Data of specific soil columns were calculated as weighted mean of sampled depth intervals (except for soil organic carbon stocks). Abbrev.: abbreviation, Min: minimum, SD: standard deviation, Max: maximum, IE: ion equivalent.

| Description | Abbrev. | Min | SD | | Median | | Mean | | Max | |  |
| --- | --- | --- | --- | --- | --- | --- | --- | --- | --- | --- | --- |
| Dynamic soil properties |  |  | |  | |  | |  | |  | |
| pH H_2_O | pH_H20_ | 4.20 | | 0.18 | | 4.65 | | 4.64 | | 4.98 | |
| pH KCl | pH_KCl_ | 3.60 | | 0.11 | | 3.83 | | 3.82 | | 4.03 | |
| Effective cation exchange capacity | CECeff | 30.52 | | 19.68 | | 52.55 | | 55.97 | | 115.20 | |
| Na [µmol_c_ g^-1^] | IE Na | 0.10 | | 0.12 | | 0.21 | | 0.24 | | 0.54 | |
| K [µmol_c_ g^-1^] | IE K | 0.75 | | 0.41 | | 1.36 | | 1.40 | | 2.39 | |
| Mg [µmol_c_ g^-1^] | IE Mg | 0.44 | | 0.76 | | 1.28 | | 1.40 | | 4.19 | |
| Ca [µmol_c_ g^-1^] | IE Ca | 0.76 | | 2.51 | | 2.69 | | 3.42 | | 9.93 | |
| Mn [µmol_c_ g^-1^] | IE Mn | 0.16 | | 0.54 | | 0.71 | | 0.82 | | 2.14 | |
| Fe [µmol_c_ g^-1^] | IE Fe | 0.11 | | 0.39 | | 0.47 | | 0.50 | | 2.11 | |
| Al [µmol_c_ g^-1^] | IE Al | 23.24 | | 17.70 | | 40.84 | | 46.45 | | 102.00 | |
| H [µmol_c_ g^-1^] | IE H | 0.83 | | 0.84 | | 1.60 | | 1.77 | | 4.58 | |
| Base saturation [%] | BS | 3.48 | | 4.90 | | 9.75 | | 10.96 | | 25.62 | |
| Exchangeable Acidity [%] | EA | 24.80 | | 18.64 | | 43.90 | | 49.54 | | 108.80 | |
| Total organic nitrogen [mass-%] | N_t_ | 0.07 | | 0.07 | | 0.13 | | 0.14 | | 0.37 | |
| Total organic carbon [mass-%] | C_t_ | 1.28 | | 1.30 | | 2.13 | | 2.51 | | 7.29 | |
| Soil organic carbon stocks [t ha^-1^] | SOCstocks | 1.14 | | 2.37 | | 3.28 | | 4.14 | | 10.91 | |
| CN ratio | CN | 13.48 | | 1.91 | | 17.65 | | 17.40 | | 21.85 | |
|  |  |  | |  | |  | |  | |  | |
| Stable soil properties |  |  | |  | |  | |  | |  | |
| Bulk Density [g cm^-3^] | BD | 0.69 | | 0.16 | | 0.96 | | 0.99 | | 1.46 | |
| Coarse Material [%] | CM | 0.00 | | 11.37 | | 5.80 | | 10.07 | | 46.50 | |
| Coarse-sized sand [%] | csa | 4.10 | | 5.75 | | 14.58 | | 15.09 | | 26.90 | |
| Medium-sized sand [%] | msa | 3.25 | | 4.45 | | 13.92 | | 13.23 | | 20.95 | |
| Fine-sized sand [%] | fsa | 1.43 | | 1.45 | | 5.58 | | 5.28 | | 7.05 | |
| Very fine-sized sand [%] | vfsa | 4.50 | | 2.68 | | 9.55 | | 9.03 | | 13.98 | |
| Coarse-sized silt [%] | csi | 6.98 | | 5.54 | | 13.55 | | 15.52 | | 26.92 | |
| Medium-sized silt [%] | msi | 8.08 | | 3.92 | | 11.70 | | 12.98 | | 24.40 | |
| Fine-sized silt [%] | fsi | 4.83 | | 2.04 | | 7.60 | | 7.89 | | 14.42 | |
| Clay [%] | clay | 12.75 | | 3.10 | | 21.25 | | 21.02 | | 26.00 | |
| Silt sum [%] | si_sum_ | 23.75 | | 9.55 | | 34.25 | | 36.41 | | 64.00 | |
| Sand sum [%] | sa_sum_ | 13.25 | | 10.24 | | 45.00 | | 42.58 | | 56.00 | |

**Supplement. Table 4.** Laboratory analysis data for all 27 CSPs and 0-30 cm soil depth columns. All elements are exchangeable elements and are shown in ion equivalents. Soil texture classes are shown according to ^71^. Data of specific soil columns were calculated as weighted mean of sampled depth intervals (except for soil organic carbon stocks). Abbrev.: abbreviation, Min: minimum, SD: standard deviation, Max: maximum, IE: ion equivalent.

| Description | Abbrev. | Min | SD | | Median | | Mean | | Max | |  |
| --- | --- | --- | --- | --- | --- | --- | --- | --- | --- | --- | --- |
| Dynamic soil properties |  |  | |  | |  | |  | |  | |
| pH H_2_O | pH_H20_ | 4.30 | | 0.17 | | 4.65 | | 4.66 | | 5.02 | |
| pH KCl | pH_KCl_ | 3.70 | | 0.09 | | 3.82 | | 3.84 | | 4.02 | |
| Effective cation exchange capacity | CECeff | 29.95 | | 17.34 | | 49.32 | | 52.39 | | 99.23 | |
| Na [µmol_c_ g^-1^] | IE Na | 0.10 | | 0.12 | | 0.20 | | 0.24 | | 0.56 | |
| K [µmol_c_ g^-1^] | IE K | 0.73 | | 0.35 | | 1.25 | | 1.30 | | 2.22 | |
| Mg [µmol_c_ g^-1^] | IE Mg | 0.36 | | 0.65 | | 1.20 | | 1.22 | | 3.76 | |
| Ca [µmol_c_ g^-1^] | IE Ca | 0.59 | | 1.83 | | 2.05 | | 2.63 | | 7.69 | |
| Mn [µmol_c_ g^-1^] | IE Mn | 0.12 | | 0.44 | | 0.54 | | 0.65 | | 1.83 | |
| Fe [µmol_c_ g^-1^] | IE Fe | 0.11 | | 0.29 | | 0.37 | | 0.40 | | 1.58 | |
| Al [µmol_c_ g^-1^] | IE Al | 23.55 | | 15.99 | | 40.18 | | 44.38 | | 89.18 | |
| H [µmol_c_ g^-1^] | IE H | 0.75 | | 0.63 | | 1.47 | | 1.60 | | 3.35 | |
| Base saturation [%] | BS | 3.25 | | 4.08 | | 9.65 | | 9.83 | | 22.35 | |
| Exchangeable Acidity [%] | EA | 24.93 | | 16.64 | | 42.63 | | 47.03 | | 94.20 | |
| Total organic nitrogen [mass-%] | N_t_ | 0.06 | | 0.06 | | 0.10 | | 0.12 | | 0.30 | |
| Total organic carbon [mass-%] | C_t_ | 1.01 | | 1.07 | | 1.67 | | 2.01 | | 5.90 | |
| Soil organic carbon stocks [t ha^-1^] | SOCstocks | 1.20 | | 2.78 | | 3.90 | | 4.88 | | 13.39 | |
| CN ratio | CN | 13.55 | | 1.76 | | 16.72 | | 16.65 | | 19.80 | |
|  |  |  | |  | |  | |  | |  | |
| Stable soil properties |  |  | |  | |  | |  | |  | |
| Bulk Density [g cm^-3^] | BD | 0.80 | | 0.15 | | 1.00 | | 1.03 | | 1.46 | |
| Coarse Material [%] | CM | 0.00 | | 14.19 | | 6.43 | | 12.66 | | 61.00 | |
| Coarse-sized sand [%] | csa | 3.77 | | 6.26 | | 14.45 | | 15.35 | | 29.10 | |
| Medium-sized sand [%] | msa | 3.30 | | 4.34 | | 14.05 | | 13.18 | | 21.17 | |
| Fine-sized sand [%] | fsa | 1.45 | | 1.41 | | 5.48 | | 5.14 | | 7.20 | |
| Very fine-sized sand [%] | vfsa | 4.57 | | 2.61 | | 9.23 | | 8.83 | | 13.35 | |
| Coarse-sized silt [%] | csi | 6.05 | | 5.65 | | 14.03 | | 15.27 | | 26.32 | |
| Medium-sized silt [%] | msi | 8.55 | | 3.95 | | 11.77 | | 12.99 | | 24.87 | |
| Fine-sized silt [%] | fsi | 5.43 | | 2.02 | | 7.40 | | 7.90 | | 13.98 | |
| Clay [%] | clay | 12.17 | | 3.31 | | 21.67 | | 21.36 | | 27.00 | |
| Silt sum [%] | si_sum_ | 22.50 | | 9.88 | | 34.50 | | 36.17 | | 64.67 | |
| Sand sum [%] | sa_sum_ | 13.17 | | 10.40 | | 43.67 | | 42.45 | | 56.33 | |

**Supplement. Table 5.** Laboratory analysis data for all 27 CSPs and 0-40 cm soil depth columns. All elements are exchangeable elements and are shown in ion equivalents. Soil texture classes are shown according to ^71^. Data of specific soil columns were calculated as weighted mean of sampled depth intervals (except for soil organic carbon stocks). Abbrev.: abbreviation, Min: minimum, SD: standard deviation, Max: maximum, IE: ion equivalent.

| Description | Abbrev. | Min | SD | | Median | | Mean | | Max | |  |
| --- | --- | --- | --- | --- | --- | --- | --- | --- | --- | --- | --- |
| Dynamic soil properties |  |  | |  | |  | |  | |  | |
| pH H_2_O | pH_H20_ | 4.35 | | 0.17 | | 4.69 | | 4.69 | | 5.04 | |
| pH KCl | pH_KCl_ | 3.71 | | 0.09 | | 3.81 | | 3.84 | | 4.01 | |
| Effective cation exchange capacity | CECeff | 29.61 | | 15.68 | | 47.20 | | 49.94 | | 88.98 | |
| Na [µmol_c_ g^-1^] | IE Na | 0.10 | | 0.13 | | 0.21 | | 0.24 | | 0.58 | |
| K [µmol_c_ g^-1^] | IE K | 0.74 | | 0.31 | | 1.21 | | 1.24 | | 2.01 | |
| Mg [µmol_c_ g^-1^] | IE Mg | 0.36 | | 0.60 | | 1.09 | | 1.17 | | 3.54 | |
| Ca [µmol_c_ g^-1^] | IE Ca | 0.51 | | 1.46 | | 1.76 | | 2.22 | | 6.53 | |
| Mn [µmol_c_ g^-1^] | IE Mn | 0.10 | | 0.38 | | 0.43 | | 0.55 | | 1.59 | |
| Fe [µmol_c_ g^-1^] | IE Fe | 0.09 | | 0.25 | | 0.32 | | 0.35 | | 1.30 | |
| Al [µmol_c_ g^-1^] | IE Al | 23.63 | | 14.69 | | 38.90 | | 42.66 | | 80.45 | |
| H [µmol_c_ g^-1^] | IE H | 0.74 | | 0.56 | | 1.44 | | 1.54 | | 2.89 | |
| Base saturation [%] | BS | 3.46 | | 3.80 | | 9.15 | | 9.45 | | 21.11 | |
| Exchangeable Acidity [%] | EA | 24.90 | | 15.22 | | 40.90 | | 45.10 | | 84.72 | |
| Total organic nitrogen [mass-%] | N_t_ | 0.05 | | 0.05 | | 0.09 | | 0.10 | | 0.25 | |
| Total organic carbon [mass-%] | C_t_ | 0.84 | | 0.89 | | 1.40 | | 1.68 | | 4.90 | |
| Soil organic carbon stocks [t ha^-1^] | SOCstocks | 1.25 | | 3.04 | | 4.38 | | 5.39 | | 15.22 | |
| CN ratio | CN | 13.39 | | 1.76 | | 15.81 | | 15.84 | | 19.62 | |
|  |  |  | |  | |  | |  | |  | |
| Stable soil properties |  |  | |  | |  | |  | |  | |
| Bulk Density [g cm^-3^] | BD | 0.81 | | 0.15 | | 1.06 | | 1.06 | | 1.46 | |
| Coarse Material [%] | CM | 0.00 | | 16.81 | | 10.12 | | 17.11 | | 68.25 | |
| Coarse-sized sand [%] | csa | 3.88 | | 6.19 | | 13.60 | | 15.38 | | 28.32 | |
| Medium-sized sand [%] | msa | 3.38 | | 4.42 | | 14.19 | | 13.25 | | 20.80 | |
| Fine-sized sand [%] | fsa | 1.49 | | 1.36 | | 5.36 | | 5.13 | | 7.20 | |
| Very fine-sized sand [%] | vfsa | 4.66 | | 2.64 | | 9.30 | | 8.76 | | 13.30 | |
| Coarse-sized silt [%] | csi | 6.01 | | 5.87 | | 13.84 | | 15.18 | | 26.49 | |
| Medium-sized silt [%] | msi | 8.89 | | 3.94 | | 11.59 | | 13.02 | | 24.95 | |
| Fine-sized silt [%] | fsi | 5.53 | | 1.94 | | 7.73 | | 7.92 | | 13.76 | |
| Clay [%] | clay | 11.88 | | 3.24 | | 21.75 | | 21.38 | | 26.75 | |
| Silt sum [%] | si_sum_ | 23.38 | | 10.00 | | 34.12 | | 36.11 | | 64.75 | |
| Sand sum [%] | sa_sum_ | 13.38 | | 10.33 | | 44.00 | | 42.49 | | 56.50 | |

**Supplement. Table 6.** Laboratory analysis data for all 27 CSPs and 0-50 cm soil depth columns. All elements are exchangeable elements and are shown in ion equivalents. Soil texture classes are shown according to ^71^. Data of specific soil columns were calculated as weighted mean of sampled depth intervals (except for soil organic carbon stocks). Abbrev.: abbreviation, Min: minimum, SD: standard deviation, Max: maximum, IE: ion equivalent.

| Description | Abbrev. | Min | SD | | Median | | Mean | | Max | |  |
| --- | --- | --- | --- | --- | --- | --- | --- | --- | --- | --- | --- |
| Dynamic soil properties |  |  | |  | |  | |  | |  | |
| pH H_2_O | pH_H20_ | 4.38 | | 0.17 | | 4.72 | | 4.71 | | 5.07 | |
| pH KCl | pH_KCl_ | 3.69 | | 0.10 | | 3.81 | | 3.84 | | 4.01 | |
| Effective cation exchange capacity | CECeff | 28.61 | | 14.46 | | 45.84 | | 47.97 | | 82.28 | |
| Na [µmol_c_ g^-1^] | IE Na | 0.10 | | 0.13 | | 0.19 | | 0.24 | | 0.61 | |
| K [µmol_c_ g^-1^] | IE K | 0.73 | | 0.29 | | 1.19 | | 1.21 | | 1.83 | |
| Mg [µmol_c_ g^-1^] | IE Mg | 0.36 | | 0.60 | | 1.01 | | 1.17 | | 3.49 | |
| Ca [µmol_c_ g^-1^] | IE Ca | 0.45 | | 1.28 | | 1.64 | | 2.01 | | 6.08 | |
| Mn [µmol_c_ g^-1^] | IE Mn | 0.09 | | 0.33 | | 0.36 | | 0.49 | | 1.44 | |
| Fe [µmol_c_ g^-1^] | IE Fe | 0.09 | | 0.22 | | 0.29 | | 0.31 | | 1.16 | |
| Al [µmol_c_ g^-1^] | IE Al | 22.64 | | 13.71 | | 37.46 | | 41.04 | | 74.64 | |
| H [µmol_c_ g^-1^] | IE H | 0.73 | | 0.54 | | 1.43 | | 1.53 | | 2.88 | |
| Base saturation [%] | BS | 4.06 | | 3.91 | | 8.74 | | 9.50 | | 21.07 | |
| Exchangeable Acidity [%] | EA | 23.90 | | 14.18 | | 39.70 | | 43.37 | | 78.54 | |
| Total organic nitrogen [mass-%] | N_t_ | 0.05 | | 0.04 | | 0.08 | | 0.09 | | 0.22 | |
| Total organic carbon [mass-%] | C_t_ | 0.71 | | 0.76 | | 1.21 | | 1.44 | | 4.18 | |
| Soil organic carbon stocks [t ha^-1^] | SOCstocks | 1.64 | | 3.23 | | 4.96 | | 5.91 | | 16.43 | |
| CN ratio | CN | 12.77 | | 1.74 | | 14.75 | | 15.12 | | 18.92 | |
|  |  |  | |  | |  | |  | |  | |
| Stable soil properties |  |  | |  | |  | |  | |  | |
| Bulk Density [g cm^-3^] | BD | 0.81 | | 0.15 | | 1.08 | | 1.08 | | 1.46 | |
| Coarse Material [%] | CM | 0.00 | | 18.51 | | 15.82 | | 20.12 | | 72.60 | |
| Coarse-sized sand [%] | csa | 4.06 | | 6.47 | | 15.13 | | 15.65 | | 29.72 | |
| Medium-sized sand [%] | msa | 3.60 | | 4.28 | | 14.12 | | 13.12 | | 20.78 | |
| Fine-sized sand [%] | fsa | 1.55 | | 1.46 | | 5.29 | | 5.15 | | 8.37 | |
| Very fine-sized sand [%] | vfsa | 4.59 | | 2.65 | | 9.22 | | 8.71 | | 13.43 | |
| Coarse-sized silt [%] | csi | 5.63 | | 5.93 | | 13.28 | | 15.21 | | 26.27 | |
| Medium-sized silt [%] | msi | 8.79 | | 3.86 | | 11.59 | | 13.02 | | 24.64 | |
| Fine-sized silt [%] | fsi | 5.28 | | 2.01 | | 7.68 | | 7.95 | | 13.69 | |
| Clay [%] | clay | 11.70 | | 3.35 | | 21.70 | | 21.21 | | 26.40 | |
| Silt sum [%] | si_sum_ | 22.30 | | 10.04 | | 33.70 | | 36.17 | | 64.40 | |
| Sand sum [%] | sa_sum_ | 13.90 | | 10.38 | | 43.80 | | 42.58 | | 60.00 | |

**Supplement. Table 7.** Descriptive statistics of four terrain attributes of 27 Comparative Study Plots. Min: minimum, SD: standard deviation, Max: maximum.

| Terrain attributes | Min | SD | Median | Mean | Max |
| --- | --- | --- | --- | --- | --- |
| Slope | 13.78 | 7.99 | 35.00 | 33.78 | 47.14 |
| Northness | -0.99 | 0.72 | -0.06 | -0.08 | 0.99 |
| Eastness | -0.99 | 0.69 | -0.07 | -0.18 | 0.99 |
| Elevation | 251 | 168 | 569 | 547 | 903 |
